# HP-NAP of *Helicobacter pylori*: The Power of the Immunomodulation

**DOI:** 10.3389/fimmu.2022.944139

**Published:** 2022-06-29

**Authors:** Gaia Codolo, Sara Coletta, Mario Milco D’Elios, Marina de Bernard

**Affiliations:** ^1^ Department of Biology, University of Padova, Padova, Italy; ^2^ Department of Experimental and Clinical Medicine, University of Firenze, Firenze, Italy

**Keywords:** HP-NAP, *Helicobacter pylori*, inflammation, allergy, cancer, therapy

## Abstract

The miniferritin HP-NAP of *Helicobacter pylori* was originally described as a neutrophil-activating protein because of the capacity to activate neutrophils to generate oxygen radicals and adhere to endothelia. Currently, the main feature for which HP-NAP is known is the ability to promote Th1 responses and revert the immune suppressive profile of macrophages. In this review, we discuss the immune modulating properties of the protein regarding the *H. pylori* infection and the evidence that support the potential clinical application of HP-NAP in allergy and cancer immunotherapy.

## Introduction

Bacteria have two types of ferritin-like molecules, the heme binding bacterioferritins (Bfr) and the non-heme binding bacterial ferritins (Ftn) (1, 2). Both are composed of 24 identical or similar subunits that form a roughly spherical protein containing a large hollow centre that acts as an iron-storage cavity with the capacity to accommodate up to 4000 iron atoms.

In 1992, Almirón and colleagues discovered a starvation-inducible protein that was strongly bound to chromosomal DNA in starved cultures of *Escherichia coli*. The protein was called Dps, as in DNA-binding protein from starved cells (3). Later, *in vivo*, and *in vitro* assays showed that Dps protected DNA during oxidative stress, by sequestering iron and by physically binding the DNA (4), although the latter activity was not demonstrated for all Dps, subsequently identified (5). Dps proteins are ubiquitous in bacteria and, to date, 76 members have been discovered in 57 organisms (6). Their sequence closeness to members of the bacterial ferritin family (7) suggested that Dps represent a new type of ferritin that takes part in a general prokaryotic approach for tackling oxidative stress. In 1998 the first crystal structure of a Dps protein was published (8). The structure proved that Dps is an analogue of ferritins. Dps monomers have essentially the same protein fold (four helix bundle) as the ferritin monomer, and they pack in a dodecameric hollow sphere which closely resembles the packing of ferritin monomers. According to their size which is smaller than that of Bfr and Ftn, Dps can store 500 atoms of iron (9).

Several names and abbreviations have been used to describe miniferritins, depending on the biochemical feature that was being studied: the most common are Dps, for their DNA-binding properties, which is often used interchangeably with miniferritin, and NAP (from neutrophil-activating protein), a term used for the first time referring to the miniferritin of *Helicobacter pylori* because of its capacity to activate neutrophils to produce oxygen radicals and adhere to endothelia (10).

The discovery that miniferritins had an impact on the function of host immune cells besides their role in protecting bacterial DNA from oxidizing radicals, has given the impetus to numerous studies on Dps proteins produced by pathogenic bacteria, such as *Borrelia burgdorferi* (NapA), *Treponema pallidum* (TpF1), *Helicobacter cinaedi* (CAIP). What emerged is that Dps proteins are major determinants in the pathogenesis of chronic inflammatory diseases because of a robust immune modulatory activity (11–14). Among the miniferritins produced by pathogenic bacteria, the most studied is certainly NAP, also called HP-NAP, produced by *H. pylori*.

This minireview summarizes the current state of knowledge on HP-NAP. We address the biological features of this Dps, highlighting the ability of promoting inflammation and dictating the profile of the adaptive immune response, as crucial in the pathogenesis of *H. pylori*-associated diseases. On the other hand, we also emphasize that it is because of its powerful and specific action on the immune system that HP-NAP has a significant potential utility in clinical practice.

## HP-NAP in *H. pylori* Infection

*H. pylori* infection is mostly acquired during childhood and often persists for life in the infected host. Depending on geographical region and economic development, the prevalence of *H. pylori* infection in adults has been found to range from 24% to 73% among populations, with a global prevalence estimate of around 50% (15). Although most infected individuals remain asymptomatic, bacteria colonization of the gastric mucosa may cause the development of various clinical conditions such as peptic ulcers, chronic gastritis and gastric adenocarcinomas, and mucosa-associated lymphoid tissue lymphomas (16). The common feature that underlies *H. pylori*-associated disorders is the generation of an inflammatory milieu that the bacterial infection elicits in the gastric mucosa. The strong recruitment of neutrophils, monocytes/macrophages, but most of all, T helper 1 (Th1) lymphocytes whose homing in the inflamed tissue is needed to potentiate the killing potential of macrophages, one would expect to be the best arsenal to fight the bacterium. On the contrary, if left untreated, the infection persists and the inflammatory status that becomes chronic lays the foundation for the development of severe diseases.

**Figure 1:**
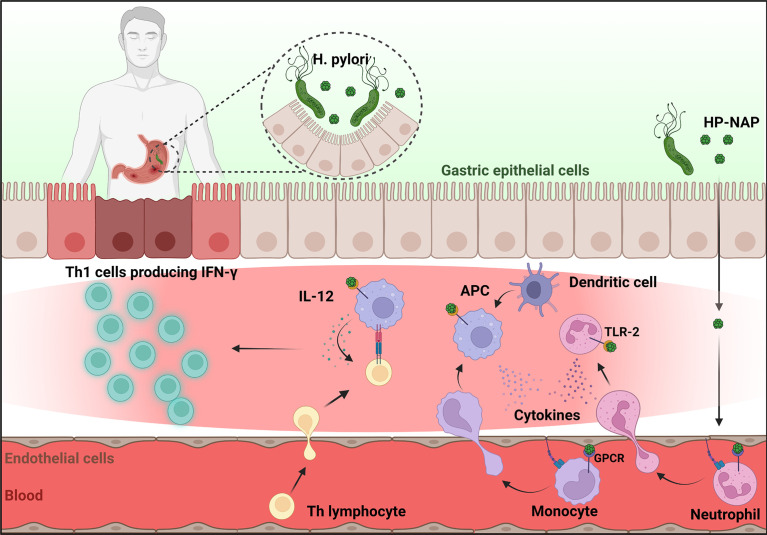
HP-NAP activity in the context of *H. pylori* infection. Once released by *H. pylori* in the stomach lumen, HP-NAP crosses the gastric epithelial cell layer and the endothelium. Bound to the luminal face of the latter, it directly stimulates leukocytes to adhere and extravasate. In addition, HP-NAP activates recruited neutrophils and monocytes to secrete cytokines that further promote inflammation, and stimulates monocytes/macrophages and dendritic cells (antigen presenting cells, APC) to release of IL-12 which drives the differentiation of T helper cells towards the IFN-γ producing Th1 phenotype. Figure created with BioRender.com.

Among several virulence factors which cooperate in promoting and maintaining inflammation, HP-NAP is probably the most active. Released by the bacterium in proximity to the gastric epithelial monolayer, HP-NAP can cross the epithelium and activate monocytes/macrophages and mast cells which represent the first line of defense, to release pro-inflammatory cytokines, i.e. TNF-α, IL-6, IL-12 and IL-23 (17, 18). HP-NAP also increases the synthesis of tissue factor (TF) and the secretion of the inhibitor-2 of the plasminogen activator in mononuclear cells (19). The coordinate expression of pro-coagulant and antifibrinolytic activities is expected to favor fibrin deposition and contribute to the inflammatory reaction elicited by *H. pylori* in the gastric mucosa. Once in the stomach wall, HP-NAP directly promotes the recruitment of leukocytes with a path resembling that adopted by the chemokine CXCL8 (20): following transcytosis through endothelial cells, a sizable amount of HP-NAP remains bound to the luminal face of the endothelium (**Figure 1**). How the luminal surface presentation of the protein occurs remains an open issue, but in this form HP-NAP encounters rolling leukocytes, up-regulates the expression of β2 integrins and induces a conformational change of these adhesion receptors, resulting in an increased affinity of them for the endothelial partner (21). This event, which is crucial for the tight adhesion of leukocytes to the endothelium, precedes extravasation. Under HP-NAP stimulation, recruited cells release pro-inflammatory cytokines and chemokines that contribute to the maintenance of inflammation by further recruiting additional neutrophils, monocytes, and lymphocytes (18, 22, 23). Several studies suggest that HP-NAP may interact with at least two receptors on the plasma membrane of leukocytes. The engagement of Toll-like receptor (TLR)-2 (18) is crucial for the production of cytokines, whereas the interaction with a G protein-coupled receptor is mainly linked to burst activation, adhesion, and chemotaxis of leukocytes (22). The evidence that the latter effects are abrogated by inhibiting p38-MAPK, suggested a role for the kinase in the signaling cascade (21, 24).

Despite the pro-inflammatory role of HP-NAP is established, the deletion of the *napA* gene does not abrogate the capacity of *H. pylori* to stimulate the production of TNF-α, IL-6, and CXCL8 by mononuclear cells, suggesting that other factors than HP-NAP are involved. On the contrary, bacteria which do not produce HP-NAP are unable to elicit the release of the Th1-polarizing cytokine IL-12 by the same cells, an event that occurs following the engagement of TLR-2 by the miniferritin (18).

*In vivo* in the antrum *H. pylori* infection causes a predominant activation of Th1 cells with production of IFN-γ and elevated expression of IL-12, IL-18, IL-17 and TNF-α (25–28). A considerable number of Th cells in the stomach mucosa of *H. pylori*-infected individuals display significant proliferation in response to HP-NAP (18, 25). According to the evidence that HP-NAP can create an IL-12-rich environment, antigen-specific gastric Th cells produce large amounts of IFN- γ and TNF-α and have a powerful cytotoxic activity in response to HP-NAP stimulation, indicating a polarized Th1/T cytotoxic 1 (Tc1) effector phenotype (18).

Collectively, these findings show that the *in vitro* and *in vivo* actions of HP-NAP are highly correlated and identify the bacterial protein as responsible for driving the Th response in the gastric antrum of patients affected by *H. pylori*. The skewing of the gastric T-cell response towards a Th1 profile, characterized by huge IFN-γ production and activation of a cytolytic program, is expected to lead to gastric damage (**Figure 1**). Moreover, the high levels of TF, IFN-γ, and TNF-α might result in procoagulant activity and in gastric functional alteration, such as increased gastrin secretion and pepsinogen release, respectively (25).

## HP-NAP as Therapeutic Tool

In view of the evidence that HP-NAP possesses a unique capacity to modulate the immune response, numerous researchers have been motivated to verify the application potential of the miniferritin as therapeutic agent. *In vivo* studies using a recombinant form of HP-NAP has been carried out in mouse model of diseases where a Th2 response is detrimental or where the induction of a Th1 and Tc1 cytotoxic immune response is beneficial, such as allergy and cancer.

### Th2 Responses

Allergic disorders, (i.e., allergic rhinitis, asthma and atopic dermatitis-AD) are Th2-mediated inflammatory diseases characterized by local infiltration of eosinophils and elevated allergen-specific IgE serum level (29, 30).

The administration of HP-NAP in a mouse model of ovalbumin (OVA)-induced allergic asthma, revealed the potent inhibitory effect of the protein on the airway eosinophil infiltration and on the Th2 bronchial inflammation, resulting in a great reduction of total serum IgE paralleled by the increase of IL-12 plasma levels (31). A similar effect was achieved in the same mouse model by injecting a plasmid encoding a protein chimera formed by HP-NAP and a soluble form of IL-4 receptor a chain, working as decoy receptor to block the IL-4 released by eosinophils and Th2 cells (32), and after orally administrating spores of *Bacillus subtilis* as a vehicle to deliver HP-NAP fused to the cholera toxin B subunit, widely used to induce peripheral immunological tolerance to co-administered antigens (33).

**Figure 2:**
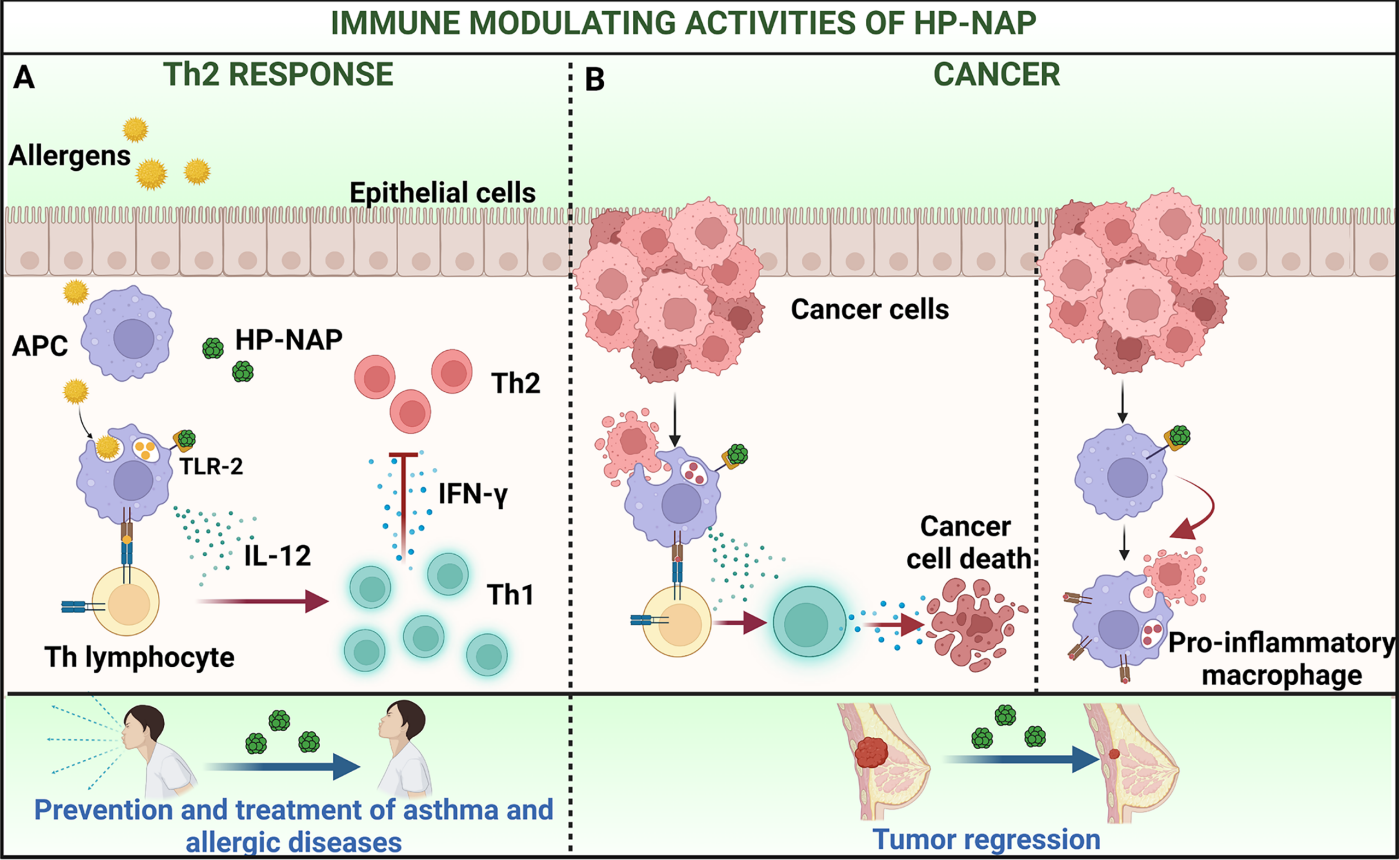
Immune modulating activities of HP-NAP applied to the treatment of allergy and cancer. **(A)** The delivery of pollen allergens to sub-epithelial APC that initiates the priming of T helper 2 (Th2) cells is a key step in the immunopathology of allergy. Treatment with HP-NAP stimulates APC to secrete IL-12 which mediate the skewing of Th2 lymphocytes towards a Th1 profile. This impacts on the allergic cascade and ameliorates the subsequent symptoms. **(B)** HP-NAP can potentiate weak natural Th1 responses, that *per se* are unable to exert protection against tumors (left) and shift the profile of macrophages from pro-oncogenic to pro-inflammatory and anti-tumoral (right). This results in a regression of tumor mass. Figure created with BioRender.com.

The capability of HP-NAP to counteract the Th2 immune responses was confirmed in a mouse model of AD (34). AD is characterized by an imbalance between Th1 and Th2 cells which results in increased production of IL-4 and IgE, and local recruitment of eosinophils (35). Intra peritoneal injection of HP-NAP significantly attenuated the secretion of IgE and IL-4 and alleviated the AD symptoms, such as erythema and swelling (**Figure 2A**).

Th2 cells not only regulate allergic disorders but are also involved in the immune response to helminth infections (36). Treatment of mice infected with the intestinal parasite *Trichinella spiralis* with HP-NAP resulted in a consistent reduction of the type 2 immune response, as revealed by the reduced eosinophil infiltration and the drop of IgE serum levels (37).

### Cancer

Cancer immunotherapy has revolutionized the field of oncology by prolonging survival of patients with rapidly fatal cancers (38). Among the variety of strategies that have become routine in the clinical practice there is the induction of Th1/Tc1 immune response with massive IFN-γ production (39). Based on the capacity to generate an IL-12-enriched environment promoting the differentiation of Th1 cells, the possibility that HP-NAP might be able to elicit an anti-tumor response, was worth investigating.

The first study, carried out in an orthotopic model of bladder cancer, revealed that the local administration of HP-NAP, by eliciting a potent Th1/Tc1 response, counteracted tumor growth and reduced vascularization of the mass due to the anti-angiogenic activity of IFN-γ (40). Notably, while the administration of Bacillus Calmette-Guérin (BCG), gold standard treatment for non-muscle-invasive bladder cancer, resulted in a strong hematuria, a condition often associated with the therapy, none of the HP-NAP-treated animals showed a macroscopic alteration of the urine aspect. Similar results were obtained in mouse models of hepatoma and sarcoma in which the protein was administered as chimera, fused with the maltose binding protein (rMBP-NAP) (41). The evidence that IFN-γ^+^ T cells were not produced, and tumor growth was not inhibited in TLR-2-knock-out mice following administration of HP-NAP (40) or by co-administrating rMBP-NAP and a TLR-2 blocking antibody (41), confirmed the *in vitro* finding suggesting the essential role of the immune receptor for the HP-NAP activity (18).

In a work by Mohabati Mobarez et al. (42), HP-NAP was loaded in chitosan nanoparticles (Chi-rNAP) and applied in a mouse model of breast cancer. The Chi-rNAP formulation strongly affected tumor growth, with an efficacy superior to that of the recombinant protein alone, in accordance to the fact that chitosan nanoparticles, by activating the antigen presenting cells, act as adjuvants (43).

Due to the ability to link the innate with the adaptive immune response, TLR agonists are highly promising as adjuvants in vaccines against life-threatening and complex diseases such as cancer. The possibility of using HP-NAP as adjuvant for cancer treatment was explored by some studies in which the protein was expressed in oncolytic viruses (OVs). The capacity of OVs to selectively replicate in tumor cells leading to cell death makes OVs promising agents for cancer therapy (44). In a neuroendocrine cancer mouse model, the intratumoral injection of OVs expressing HP-NAP improved the animal survival and increased the plasma level of the p40 subunit of IL-12 (45). Using an adenoviral vector encoding HP-NAP, it was demonstrated that the protein promotes the maturation of dendritic cells, both *in vitro* and *in vivo*. Dendritic cells matured by vector-encoded HP-NAP secrete high level of IL-12, and in accordance have the capacity to induce antigen-specific T cell expansion with a predominant Th1 profile. In the same line of evidence it was shown that HP-NAP *per se* promotes the maturation of dendritic cells and the activation and proliferation of cytotoxic T cells towards melanoma cells (46, 47).

An attenuated measles virus strain and vaccinia virus were engineered to express HP-NAP and both were effective in counteracting tumor growth and in improving the survival of animals with breast cancer and neuroblastoma, respectively (48, 49). CAR T cells engineering to produce HP-NAP turned out to be a very promising approach for treating solid tumors that are difficult to completely eradicate with conventional CAR T cells, due to heterogeneity in antigen expression. In mouse models of cancer, injection of CAR(NAP) T cells slowed tumor growth and increased survival rates compared to standard mice CAR T cells, regardless of target antigen or tumor type. The evidence on the safety of this approach in mice bode well for its clinical application (50).

All these studies have converged on the notion that the anti-tumor potential of HP-NAP relies on the activation and shaping of the adaptive immune response, but the possibility that HP-NAP might counteract tumor growth due to the modulation of mononuclear cells, regardless of the participation of the adaptive immunity, remained unexplored. Codolo and colleagues, taking advantage of the zebrafish model, examined the therapeutic efficacy of HP-NAP against metastatic human melanoma, limiting the observational window to 9 days post-fertilization, well before the maturation of the adaptive immunity. The study disclosed a new property of the miniferritin, namely the capacity of reverting the immune suppressive profile of macrophages, so as to counteract the tumor growth even in the absence of the acquired immune system (51).

## Concluding Remarks

Since its discovery in 1995, HP-NAP, the miniferritin produced by *H. pylori* has been under intense focus because of its remarkable ability to modulate the human immune response. *H. pylori* infection leads to an intense inflammatory response in the gastric mucosa, characterized by the infiltration of polymorphonuclear and mononuclear cells. It is assumed that HP-NAP, by cooperating to the recruitment of inflammatory cells but especially by generating a pro-inflammatory Th1 skewing environment (**Figure 1**) can make a substantial contribution to the gastric damage caused by *H. pylori* infection. In accordance, HP-NAP is one of the antigens included in the vaccine formulations currently under investigation (52).

On the other hand, the immune modulating activity of HP-NAP makes it an excellent candidate for developing new therapeutic strategies aimed at preventing and treating allergic disorders, such as bronchial asthma, rhinitis, conjunctivitis and, most importantly, at fighting malignant tumors (**Figures 2A, B**).

Whether the iron-binding ability of HP-NAP is related to the pathogenesis of *H. pylori* infection or to the immune modulating properties of the miniferritin is not clear. The bacterial protein is constitutively expressed under iron-depletion, and its expression is not regulated by the presence or absence of iron and it has no part in the metal resistance of *H. pylori* (53). Probably HP-NAP protects the bacterium from the oxidative stress produced in ferrous ion-mediated Fenton reactions, since the degree of DNA damage is much higher in the *napA* knock-out mutant strain than that in the wild-type strain (54). Moreover, iron plays an important role in generation of the quaternary structure of HP-NAP by promoting stable dimers that are crucial for the ensuing dodecamer structure (55), that is likely to be essential for the immune modulatory properties.

Although more pre-clinical studies are mandatory, the evidence of the clinical potential of HP-NAP are promising and strongly support the possibility of adopting HP-NAP as immunomodulatory agent. The immunostimulatory activity of the bacterial protein could also enhance the immunogenicity of poor immunogens, thus HP-NAP could be used as an adjuvant to be included in vaccines formulations.

## Author Contributions

MdB and MMDE conceived the article content. GC prepared the first draft. SC provided a critical review and prepared the figures. All authors contributed to the article and approved the submitted version.

## Conflict of Interest

The authors declare that the research was conducted in the absence of any commercial or financial relationships that could be construed as a potential conflict of interest.

## Publisher’s Note

All claims expressed in this article are solely those of the authors and do not necessarily represent those of their affiliated organizations, or those of the publisher, the editors and the reviewers. Any product that may be evaluated in this article, or claim that may be made by its manufacturer, is not guaranteed or endorsed by the publisher.
